# Ten-Hour Stable Noninvasive Brain-Computer Interface Realized by Semidry Hydrogel-Based Electrodes

**DOI:** 10.34133/2022/9830457

**Published:** 2022-03-10

**Authors:** Junchen Liu, Sen Lin, Wenzheng Li, Yanzhen Zhao, Dingkun Liu, Zhaofeng He, Dong Wang, Ming Lei, Bo Hong, Hui Wu

**Affiliations:** ^1^State Key Laboratory of New Ceramics and Fine Processing, School of Materials Science and Engineering, Tsinghua University, Beijing 100084, China; ^2^State Key Laboratory of Information Photonics and Optical Communications and School of Science, Beijing University of Posts and Telecommunications, Beijing 100876, China; ^3^Department of Biomedical Engineering, School of Medicine, Tsinghua University, Beijing 100084, China; ^4^School of Artificial, Beijing University of Posts and Telecommunications, Beijing 100084, China; ^5^School of Biomedical Engineering, Hainan University, Haikou 570228, China

## Abstract

Noninvasive brain-computer interface (BCI) has been extensively studied from many aspects in the past decade. In order to broaden the practical applications of BCI technique, it is essential to develop electrodes for electroencephalogram (EEG) collection with advanced characteristics such as high conductivity, long-term effectiveness, and biocompatibility. In this study, we developed a silver-nanowire/PVA hydrogel/melamine sponge (AgPHMS) semidry EEG electrode for long-lasting monitoring of EEG signal. Benefiting from the water storage capacity of PVA hydrogel, the electrolyte solution can be continuously released to the scalp-electrode interface during used. The electrolyte solution can infiltrate the stratum corneum and reduce the scalp-electrode impedance to 10 k*Ω*-15 k*Ω*. The flexible structure enables the electrode with mechanical stability, increases the wearing comfort, and reduces the scalp-electrode gap to reduce contact impedance. As a result, a long-term BCI application based on measurements of motion-onset visual evoked potentials (mVEPs) shows that the 3-hour BCI accuracy of the new electrode (77% to 100%) is approximately the same as that of conventional electrodes supported by a conductive gel during the first hour. Furthermore, the BCI system based on the new electrode can retain low contact impedance for 10 hours on scalp, which greatly improved the ability of BCI technique.

## 1. Introduction

**Figure 1:**
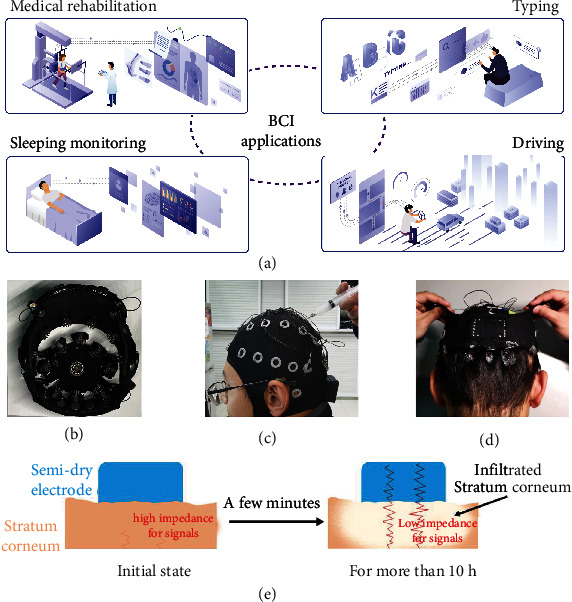
(a) Schematic diagram of BCI applications: medical rehabilitation, typing, sleeping monitoring, and driving. (b) Gel injection process for Ag/AgCl conventional electrodes on a subject using a syringe with a needle to decrease the contact impedance between the electrode and scalp. (c) After the use of a wet electrode, a large amount of conductive gel sticks to hair and electrode cap. (d) A dry electrode cap. (e) A schematic for using AgPHMS semidry electrode on scalp, low-impedance stratum corneum benefits signal transmission.

There are increasingly demands for communication between humans and computers [1–3]. The brain-computer interface (BCI) is a technique of communication based on neural activity generated by the brain and is independent of the brain's normal output pathway of peripheral nerves and muscles; the BCI is a novel communication channel without the use of traditional human-computer interaction equipment, such as a keyboard and mouse [4–8]. By collecting and analyzing electroencephalogram (EEG) signals, the BCI system provides access to a wealth of real-time brain information, including brain activity and mental states [9–11]. Because the BCI system has many advantages such as real-time interaction, accuracy, and being movement-independent, there are many high hopes for the practical applications of BCI system (Figure 1(a)), including uses in medical rehabilitation [12–15], sleeping monitoring [16–18], driving [19–23], and typing [24–28].

To ensure the practicality of EEG, a suitable electrode needs to have many key properties, such as ease of installation and clean, having long-term effectiveness and high conductivity, being comfortable to wear, and biocompatibility [29]. In many BCI applications or studies, commercial dry electrodes and wet electrodes have been widely used to collect EEG signals [30, 31]. However, commercial dry electrodes cannot be used in some applications that require high-quality signal due to the high contact impedance. And the claws on the top of electrodes make users feel uncomfortable, which also hinders the popularization of commercial dry electrodes (Figure 1(b)) [32–34]. Benefiting from the sufficiently low impedance (<10 k*Ω*), the gel-based rigid silver/silver chloride (Ag/AgCl) electrode (called a wet electrode) is used in many situations as a gold standard to sense EEG signals [35–38]. Since the stratum corneum of the scalp is infiltrated by the electrolyte in the conductive gel, the contact impedance between the scalp and electrode is significantly reduced when the wet electrode is used [39]. To ensure a good signal collecting conduction between the scalp and the electrode, a large amount of commercial conductive gel needs to be injected at the electrode-scalp interface (Figure 1(c)), which is time-consuming, uncomfortable, and may negatively affect the skin. And after use, washing off the conductive gel from the hair and electrode cap is also a time-consuming process (Figure 1(d)). The most serious problem is that the moisture in the conductive gel continuously evaporates during use. Once the electrode impedance achieved an acceptable value by injecting conductive gel, a countdown begins until the gel dries. During this period, the EEG signal quality will decrease as the impedance increases, until the available signal disappears [40–42]. Therefore, wet electrodes cannot be used for long-term applications, which severely limits the popularization of BCI technologies. In order to achieve long-term BCI applications, a long-lasting EEG electrode with low contact impedance is necessary. Recently, a lot of studies focus on how to prepare a long-lasting electrode. At present, the semidry electrode is a recognized electrode that can simultaneously meet the need for low impedance and durability. The semidry electrode combines the advantages of wet electrodes and dry electrodes and has become a new research focus of EEG signal collection electrodes [43–46]. Typically, the electrolyte volume in semidry electrode is significantly less than that of conductive gels used for the wet electrode. With such a small amount of electrolyte, semidry electrodes can infiltrate the stratum corneum and reduce the electrode-scalp contact impedance. The electrolyte in the semidry electrode is liquid, rather than the gel in the wet electrode [47–49]. Therefore, when designing a semidry electrode, the durability of the semidry electrode can be increased by increasing the volume of the water storage and meet the needs of some long-term BCI applications for long-lasting electrode. However, the semidry electrode still has some problems that must be solved. To ensure the stability of the structure, the semidry electrode with a microporous and water tank structure is rigid. This leads to discomfort that is similar to that of wearing commercially dry electrodes, and the small scalp-electrode contact surface also causes relatively high contact impedance. One of the most challenging problems is controlling the release speed of electrolyte to the scalp. If the electrolyte is released too slowly, the resistance of the stratum corneum will increase. On contrast, it will cause a short circuit between the signal channels if the release is too fast. Therefore, a comfortable gel-free electrode with a facile preparation process, high conductivity, good mechanical and electrochemical stability, long-term service, and sustained release ability of electrolyte is highly desired for a noninvasive BCI system.

Herein, we report a flexible, cost-effective, mass-produced, robust, controlled-released electrolyte and long-lasting silver-nanowire/PVA hydrogel/melamine sponge (AgPHMS) semidry electrode for noninvasive BCI systems. In the hydrogel precursor solution, we mixed NaCl/glycerol aqueous solution. Because of the water storage and liquid released capacity of PVA hydrogel, the electrolyte solution can be continuously and controlled released to the scalp-electrode interface. Benefit from the controlled-released effect, the stratum corneum impedance and contact impedance can maintain a low value for a long time without the risk of a short circuit occurring between adjacent channels. The quality of EEG signals transmitted through the low-impedance scalp is high, which helps the terminal devices to more accurately analyze brain activity (Figure 1(e)). Furthermore, the electrolyte solution is slowly released without any special liquid storage or transport structure; this increases the stability of the electrode and reduces the cost and difficult of assembling the electrode. In addition, the new electrode shows high conductivity, good flexibility, and remarkable electrochemical and mechanical stabilities, which are highly expected for the widely application of long-term noninvasive BCI systems.

## 2. Results and Discussion

### 2.1. Preparation, Micromorphology, and Elemental Analysis of AgPHMS

**Figure 2:**
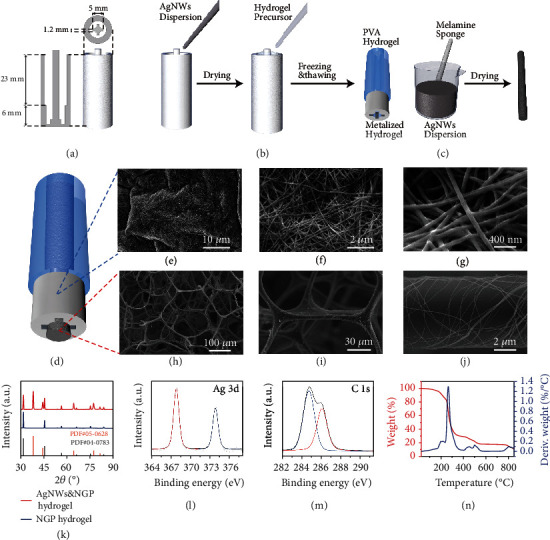
(a) Illustration of the structure of a 3D-printed mold. (b) Fabrication process of the partially metalized PVA hydrogel. (c) Fabrication process of the metalized melamine sponge. (d) Illustration of the structure of the AgPHMS semidry electrode. (e–g) SEM images of metalized PVA hydrogel. (h–j) SEM images of the metalized melamine sponge. (k) XRD spectra of metalized PVA hydrogel and the hydrogel substrate. (l) XPS peak-differentiation-imitating analysis of Ag 3d, performed on metalized PVA hydrogel. (m) XPS peak-differentiation-imitating analysis of C 1s, performed on metalized PVA hydrogel. (n) TGA (red) and differential thermal analysis (blue) of metalized PVA hydrogel.

A new EEG AgPHMS semidry electrode includes a partially metallized PVA hydrogel and a metallized sponge. The PVA hydrogel precursor was cross-linked in a designed 3D printing mold to prepare a specific shape partially metalized hydrogel (Figure 2(a)). The fabrication process of the partially metalized PVA hydrogel is shown in Figure 2(b). First, silver nanowire (AgNW) solution was dropped in the mold. After the solvent entirely evaporated, there was a silver film on the surface of the mold. Then, the PVA hydrogel precursor was dropped in the mold and cross-linked it via cycle freezing and thawing. Before the cross-link process, sodium chloride and glycerol solution were added to the hydrogel precursor to more effectively reduce the resistance of the stratum corneum by the electrolyte released in the hydrogel. Finally, a partially metalized PVA hydrogel was obtained after removing the cross-linked PVA hydrogel from the mold. Based on our previous work, [29] a commercial melamine sponge was fully immersed in prefabricated AgNWs and polyvinyl butyral (PVB) solution during a vacuum processing step to prepare a metallized sponge. After drying, a metallized sponge was obtained (Figure 2(c)). Metallized hydrogel and a sponge were assembled to make a complete electrode (Figure 2(d)). As shown in the SEM image of the freeze-dried metalized hydrogel, AgNWs are interconnected, and the AgNWs are protected by a thin layer of PVA hydrogel; this ensures the high conductive surface and durability of the partially metallized hydrogel (Figures 2(e)–2(g)). AgNWs that have a large aspect ratio can wrap around the sponge skeleton, ensuring the high conductivity and mechanical stability of the metallized sponge (Figures 2(h)–2(j)). Figure 2(k) shows the XRD spectrum of the freeze-dried metallized hydrogel and that of the hydrogel without metallization. Because NaCl crystallizes during freeze drying, there are NaCl peaks (PDF#05-0628) in the spectrum in addition to from the peaks of silver (PDF#04-0783). X-ray photoelectron spectroscopy (XPS) result of the metallized hydrogel shows characteristics of metallic silver and PVA, suggesting that a highly metalized surface is fixed and protected by a PVA polymer coating. The peak of Ag 3d consists of two metal peaks at 367.5 eV and 373.5 eV (Figure 2(l)) [50]. In the C 1s spectrum, there are two separate peaks for PVA: unoxidized carbon (C–C or C–H) and carbon with one oxygen bond (C–OH or C–O–) (Figure 2(m)) [51]. Thermogravimetric (TG) results of the freeze-dried PVA hydrogel with metallized surface from 25°C to 850°C at 10°C per minute (Figure 2(n)) prove that the PVA hydrogel with metallized surface is thermally stable when the temperature is below 100°C. XRD (Figure S1), XPS (Figure S2), TGA, and differential thermal analysis (Figure S3) of the metalized sponge suggest that the sponge was metalized and thermally stable.

### 2.2. Mechanical and Electrochemical Stabilities of a AgPHMS Semidry Electrode

**Figure 3:**
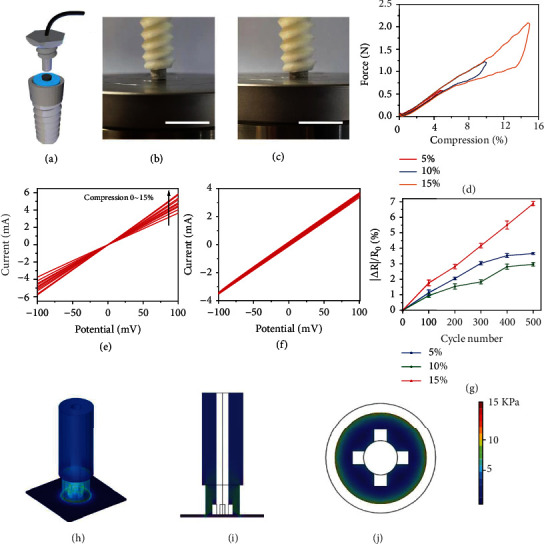
(a) Semidry electrode and hollow screw structure shell. Photographs of the mechanical stability test setup under (b) compression and (c) release. The scale bars in (b) and (c) are 2 cm. (d) Stress-strain curve of the AgPHMS semidry electrode with 5%, 10%, and 15% compression. (e) Linear sweep voltammetry (LSV) results of the AgPHMS semidry electrode for different values of compression. (f) 500 cycles of CV on the AgPHMS semidry electrode. (g) Changes of AgPHMS semidry electrode impedance in 500 compression cycle tests with 5%, 10%, and 15% compression (*n* = 3 for each compression). (h) Perspective of finite element simulation results with 10% compression. Views along the (i) *y*-axis section and (j) *z*-axis section of finite element simulation results with 10% compression.

To test and use the new AgPHMS semidry electrode, we designed and made a hollow-screw shell holder (Figure 3(a)). The electrode can be fixed in a cylindrical hole with only the top exposed, and a hollow screw can be further loaded on the test equipment and EEG electrode cap. The shell ensures stability during use and prevents the electrolyte in the hydrogel from quickly volatilizing into the air. Mechanical properties of the new electrode were tested by compressing and releasing it (Figures 3(b) and 3(c)). Because the electrode is in a PLA plastic shell and almost the entire electrode is pressed in the shell under 15% compression, we tested the stress-strain curve of the electrode with 5%, 10%, and 15% compression (Figure 3(d)). When the compression was 15%, the entire electrode was pressed into the shell, and the stress of the electrode at this point is 2.2 N. The stress of the electrode at 5% and 10% are 0.5 N and 1.0 N, respectively, which suggests that the electrode is flexible and comfortable to wear. Electroperformance is a key characteristic of EEG electrodes for signal recording. According to linear sweep voltammetry (LSV) results, when the electrode was compressed, resistance reduced from 28 *Ω* to 17 *Ω* because of the simultaneous increase in the silver density on the metallized sponge (Figure 3(e)). Under every compression, voltage and current maintain a good linear relationship, which proves that no electrochemical reaction will occur during the EEG signal transmission. The CV curve is stable during 500 potential cycles, which suggests that the electrochemical properties of the electrode will be stable during the EEG signal collection (Figure 3(f)). Since the electrode may be compressed during assembly and use, repeated compression experiments were performed on the electrode to test its long-term mechanical stability and flexibility. The self-impedance of the electrode changed no more than 2.5% after 500 cycles with a 10% compression ratio. Under more extreme conditions, the self-impedance of the electrode changed no more than 3.5% with a 5% compression ratio and no more than 7% with a 15% compression ratio (Figure 3(g)). To research the force details of hydrogel with compression, the compression state was simulated on a computer using finite element analysis. At the electrode-scalp interface, the pressure did not exceed 15 KPa with a 10% compression (Figures 3(h)–3(j)), which is comfortable for the users of this electrode.

### 2.3. BCI Applications and Biocompatibility

**Figure 4:**
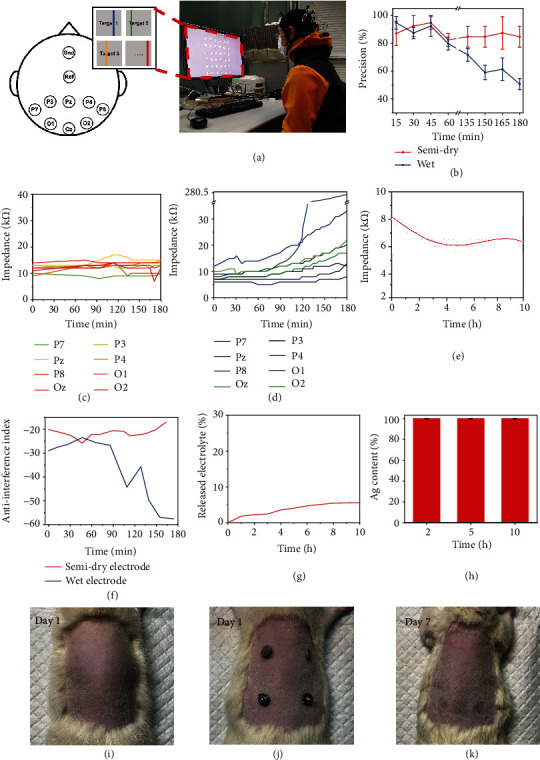
(a) Brain mapping of the AgPHMS semidry electrode EEG cap in a BCI system and typing using mVEP mapping. (b) Accuracy of typing during the first hour and the third hour using the AgPHMS semidry electrode and a commercial wet electrode (*n* = 3 for each electrode). (c) Impedance of the AgPHMS semidry electrode system on different channels. (d) The impedance of a commercial wet electrode system on different channels. (e) Ten-hour impedance data of the AgPHMS semidry electrode system. (f) Anti-interference index of the AgPHMS semidry electrode and commercial wet electrode. (g) Release of electrolyte from the AgPHMS semidry electrode. (h) Silver content on the AgPHMS semidry electrode when immersed in electrolyte (*n* = 3 for each sample). (i, j) Biocompatibility test of the AgPHMS semidry electrode using conventional Ag/AgCl gel-based electrodes as the control group. (k) Neither test triggered an allergic reaction after 7 days.

For BCI applications, three-hour motion-onset visual evoked potential (mVEP) experiments were performed according to two scenarios: using conventional wet electrodes and using AgPHMS semidry electrodes. The original EEG signals of the subjects were first recorded with an amplifier on an 8-channel electrode cap; the EEG signals were sent to the terminal computer using Wi-Fi. The participant typed the phrase “THU HELLO WORLD” by looking at a virtual keyboard including 26 English letters “A-Z” and 10 Arabic numerals “0-9” on the computer monitor. On each symbol of the virtual keyboard, there was a vertical line across at a specific frequency; this stimulated a specific EEG signal of the subject. The character that the subject is looking at can be mapped on the computer by analyzing the characteristics of the signal (Figure 4(a), Figure S4, and Supplementary Video 1). For each scenario, the participant repeated the typing experiment 8 times in three hours. During the experiment, we counted the accuracies of typing results and impedance to evaluate the performance of the electrodes. During the first hour, the average accuracy AgPHMS semidry electrode was 86.5%, and during the third hour, this value was reduced by 6% to 81%, which meant that the BCI system using AgPHMS semidry electrodes can maintain high accuracy during a long-term mVEP experiment. The accuracy of the BCI system based on wet electrodes in the first hour (92.5%) is slightly higher than that of the BCI system based on AgPHMS semidry electrodes. During the third hour, the accuracy of the subject that used wet electrodes was reduced by 34% to 61%, (Figure 4(b)). During mVEP experiments, the system impedance data of every channel was recorded. The impedance of the AgPHMS semi-dry electrode BCI system was stable at 8 ~ 14 k*Ω*, and the change rate of impedance of each channel is less than 40% during this three-hour experiment (Figure 4(c) and Figure S5a). During the first hour, the impedance of the wet electrode BCI system was 5 ~ 14 k*Ω*, which was same as that of the AgPHMS semidry one. Starting from the second hour, the impedance of some wet electrode channels such as P8, O1, O2, and Oz have an increasing trend, and the rate of increase obviously increased during the third hour (Figure 4(d)). The maximum impedance change rate of wet electrode BCI system reached 2200% which could be attributed to volatilization of moisture in the wet electrode (Figure S5b). Through a ten-hour impedance recording of channel P7 and P8, the impedance of the AgPHMS semidry electrode BCI system was stable at 6 ~ 8.5 k*Ω*, which suggests that this AgPHMS semidry electrode can be used for super long-term BCI experiments (Figure 4(e)). An anti-interference index was defined according to the steady-state visual evoked potential (SSVEP) data (Figure S6). The anti-interference of the AgPHMS semidry electrode BCI system was stable at -16~ -25. The anti-interference of the wet electrode BCI system was -23 to -28 during the first hour, -25 to -44 during the second hour, and -35 to -57 during the third hour. Generally speaking, the anti-interference of the AgPHMS semidry electrode BCI system was getting lower and lower during this three hours' SSVEP experiment (Figure 4(f)). We found that the anti-interference and impedance show similar trends in two scenario experiments. As a result, the accuracy of the wet electrode system decreased with a decrease in impedance, and the accuracy of the AgPHMS semidry electrode BCI system remained unchanged. A great AgPHMS semidry electrode can release electrolyte at a slow speed. During 10 hours of wearing, the PVA hydrogel continuously released about 5.6 wt% electrolyte, which can continuously infiltrate the stratum corneum and act as an ionic conductive agent for EEG signal gain to complement the electronic conduction of the AgNWs (Figure 4(g)). An electrolyte can ensure the low contact resistance of the BCI system during use. We soaked the electrodes in electrolyte for 10 hours to test the stability of the combination of silver nanowires and hydrogel in an environment full of NaCl/glycerol aqueous solution electrolyte. As seen in Figure 4(h), no silver was shed in the electrolyte, which is necessary for the electrode to ensure the conductivity and biosafety during the long-term using. After using the wet electrode, a lot of conductive gel sticks to the hair, but there was no such trouble with the AgPHMS semidry electrode (Figure S7). To measure the biocompatibility of the AgPHMS semi-dry electrode, we performed a 7-day patch test on two rats using two groups of symmetrically arranged electrodes: two AgPHMS semidry electrodes and two conventional Ag/AgCl gel-based electrodes (Figures 4(i) and 4(j)). Attaching the two kinds of electrodes to the rats' skin for 7 days showed no adverse effects, including erythema or complications, at the attachment sites (Figure 4(k)). Electrolyte soaked and biocompatibility test results suggest that the AgPHMS semidry electrode is compatible with skin and is suitable for long-term EEG monitoring.

## 3. Conclusion

In this work, we developed a cost-effective, easily manufacturable, flexible, robust, gel-free biocompatible, and tank-free EEG AgPHMS semidry electrode that has a controlled-released electrolyte and low impedance and can be used long term. This electrode was used to establish a reliable long-lasting noninvasive BCI system. Taking advantage of its high flexibility and controlled-released electrolyte, this electrode has the ability to maintain the impedance of the BCI system at 5-15 k*Ω* for more than 10 hours. We demonstrate the successful application of the AgPHMS electrodes in an eight-channel BCI system and a mind control typing experiment based on mVEPs. MVEP experiments show a 77%-100% accuracy for the AgPHMS semidry electrodes and this high accuracy can be maintained for more than 3 hours. As a benefit of the long-term (>10 hours) low impedance, the accuracy of the AgPHMS semidry electrodes can maintain accuracy for a long time. The new electrode has great potential for use in long-term BCI applications.

## 4. Experimental Section

### 4.1. Materials

Silver nitrate (AgNO_3_, 99.8%), copper chloride (CuCl_2_·2H_2_O, AR), sodium chloride (NaCl, 99.5%), ethyl alcohol (EG, 99.5%), polyvinylpyrrolidone (PVP, Mw = 360,000), polyvinyl butyral (PVB, Mw = 170,000), and polyvinyl alcohol (PVA, Mw = 1750) were purchased from Aladdin. Ethanol (99.7%), acetone (99.5%), and glycerol (99.0%) were purchased from Modern Oriental (Beijing) Technology Development Co., Ltd. Melamine sponge and 3D printable polylactic acid (PLA) are commercially available.

### 4.2. Synthesis of AgNWs

All of the reagents were used without further purification. 0.8 g of polyvinylpyrrolidone (PVP, Mw = 360,000) and 1.0 g of silver nitrate (AgNO_3_) were sequentially dissolved in 100 mL of absolute ethyl alcohol (EG) under magnetic stirring. After these were thoroughly dissolved, 1.6 mL of as-prepared CuCl_2_·2H_2_O (3.3 mM) EG solution was rapidly injected into the mixture and gently stirred. The mixture solution was then immersed in a preheated silicone oil bath at 130°C for 3 h. Finally, after the growth was complete, the resultant solution was cleaned three times using acetone and ethanol and centrifuged at 3000 rpm for 10 min. The resultant AgNWs were dispersed in ethanol, forming 100 mg/g AgNWs/ethanol mixture solution for further use [52].

### 4.3. Preparation of Metallized Melamine Sponge

In a typical process, a cylindrical puncher was used to punch melamine sponge into a cylinder that was 2.5 cm high and 0.2 cm in diameter. PVB (0.8 g) was dissolved in 100 mL of ethanol and stirred at 80°C for 40 min. After cooling naturally, 1.5 g of ethanol-dispersive AgNWs (100 mg/g) was added into the mixture and stirred for 10 min. The cylinder sponges were then immersed in AgNWs/PVB solution and subjected to vacuum treatment under 2000 Pa for 10 min. Metallized melamine sponge was finally obtained after natural drying.

### 4.4. Preparation of Partially Metallized NaCl-Glycerin-PVA Hydrogel

A PLA mold was 3D printed (Figure 2(a)) to obtain hydrogel with a specific shape. 1.5 g of ethanol-dispersive AgNWs (100 mg/g) was added to 100 mL of ethanol and stirred for 10 min. The AgNW solution was added to the mold until the depth of the solution in the mold reached 6 mm. This was fully dried until the ethanol in the mold was completely volatilized. PVA powder (1 g) was added to 5 mL of ultrapure water and subjected to vigorous stirring at 90°C to dissolve it entirely. 0.35 g NaCl powder (0.35 g) and glycerol (0.7 g) were added to 1 mL or ultrapure water and subjected to vigorous stirring to dissolve it entirely. The hydrogel precursor was obtained by entirely mixing the NaCl/glycerol solution with the PVA solution. After filling the mold with the hydrogel precursor solution, we stored the mold at -20°C to freeze the hydrogel precursor and then thawed it at 25°C. After three freeze-thaw cycles, the precursor was cross-linked into a hydrogel. After removing the hydrogel from the mold, we obtained a piece of partially metalized hydrogel. The metalized sponge was inserted into the hole in the middle of the partially metallization hydrogel to obtain the AgPHMS electrode.

### 4.5. Assembly of the AgPHMS Electrode and EEG Cap

The shell of the AgPHMS electrode was a 3D-printed PLA hollow screw (Figure 3(a)). The top screw can be used to fix the electrode in the inner space, and the shell can be screwed into the nut on the electrode cap. DuPont wire was used to connect the end of the metalized sponge to the signal amplifier on the electrode cap. Conductive silver paste was applied on the contact surface of the Dupont wire and the sponge. After assembly, ten AgPHMS electrodes were positioned at ten sites on the cloth EEG cap, including the eight working points of P7, P3, Pz, P4, P6, O1, Oz, O2, one reference point of Ref, and one grounding point of Gnd.

### 4.6. Micromorphological Characterization

X-ray powder diffraction data were collected using an X-ray powder diffraction (XRD) (D/max 2500, Rigaku, Japan) with Cu K*α* radiation (*λ* = 1.54178 Å). Micromorphological images were recorded using a field emission scanning electron microscope (FE-SEM, LEO-1530, Zeiss, Germany). An X-ray photoelectron spectrometer (Escalab 250Xi, Thermo Fisher, America) equipped with an Al K*α* radiation source (1487.6 eV) and a hemispherical analyzer with a pass energy of 30.00 eV was used to obtain surface element information. Thermogravimetric analysis (TGA) and differential thermal gravity analysis were performed using a thermogravimetric analyzer (STA 449 F3, Jupiter, Germany). Element concentrations were measured using an ICP-OES (iCAP 7600, Thermo Scientific, USA).

### 4.7. Mechanical and Electrochemical Properties

Mechanical properties of AgPHMS were measured using a mechanical testing machine (Z1.0 TH, Zwick, Germany) with a load sensor (Xforce HP load cell, capacity 1 kN). Mechanical stability of AgPHMS was assessed at room temperature and measured using a universal material testing machine in the cyclic compression mode. This was combined with a computer-controlled electrochemical workstation (CHI 660D, CH Instrument, China) using a two-electrode system. Measurement of electrochemical properties of AgPHMS, cyclic voltammetry (CV), and linear sweep voltammetry (LSV) were recorded using a computer-controlled electrochemical workstation (CHI 660D, CH Instrument, China).

### 4.8. FEA Simulation

The FEA simulation was performed using the mechanics module in COMSOL. Young's modulus of the hydrogel was calculated to be 58.6 kPa. For contact, 10% relative displacement was applied on the AgPH.

### 4.9. EEG Signal Acquisition

The EEG signal was collected using a wireless EEG acquisition system (NeuSen W, Boruikang, China). When wet electrodes were used, conductive gel (GT5, Greentek, China) was injected into the Ag/AgCl electrodes until the impedance was below 10 k*Ω*.

### 4.10. Motion-Onset Visual Evoked Potential (mVEP) Stimulation Paradigm

Motion-onset visual evoked potentials (mVEPs) are a well-established nonflashing visual BCI paradigm. The principle of mVEPs is that a bar that moves from right to left within a target virtual button can evoke a visual motion stimulus to the subjects.

For the mVEP (motion-onset visual evoked potential) paradigm, the visual stimuli were presented on a 23-inch LCD monitor with a resolution of 1920 × 1080 pixels and a refresh rate of 60 Hz at a viewing distance of 50 cm. 36 rectangular virtual buttons correspond to different characters (‘A' to ‘Z', ‘0' to ‘9'). The buttons are arranged in 6 columns and 6 rows (Figure S2).

Each visual motion stimulus was evoked by a moving vertical bar. The bar appears at the right border of the virtual button (stimulus onset) and moves to the left before it disappears (about 9.5 mm on the screen, taking 150 ms). Each epoch consists of six stimuli in one row/column at the same time, with 50 ms interval between two epochs. Then, twelve epochs (six row-epochs and six column-epochs of the keyboard) form a trial, which lasts 2400 ms. During one trial, assume that “Z” was the attended target. When the subject stares at the “Z” button, the 5th row-epoch and the 2nd column-epoch elicits target responses (known as N200 or mVEPs).

Subjects participated in two sessions; one was offline, and one was online. In both sessions, the subjects were required to stare at the center of the target virtual button.

1. In the offline session, a red border was used to inform the subjects which virtual button was the target before each trial. Then, after 60 trials (repeated 10 times for each character on one diagonal, for example, ‘AHOV29'), data was used to train a binary classifier for discriminating target and nontarget stimuli
2. In the online session, subjects were instructed to sequentially type the phrase ‘THU HELLO WORLD.' The previous binary classifier was used for each character, and then, the value of the target button was returned according to the moment of the target stimuli (row- and column-epoch elicit responses). When one character was mistyped, the subjects were told not to correct that character but to continue with the next. At the end of this session, the number of mistyped characters were recorded for further calculation

The software for this paradigm was developed using Python (Python Software Foundation) and Psychopy; more details of this paradigm are elaborated in our previous work [53].

These two sessions were conducted using two different types of EEG electrodes, a traditional wet electrode and a semidry electrode. Both types of electrodes used eight electrodes (P7, P8, P3, P4, Pz, O1, O2, and Oz) covering occipital and parietal areas, and the electrodes CPz and AFz were chosen as REF and GND, respectively. For the traditional wet electrode, the impedance of all of the electrodes was reduced to 10 K*Ω*. Signals were sampled at 1000 Hz. Trigger events were acquired simultaneously with stimulus onsets.

For each type of electrode, the offline session was conducted three times. Then, the classifier with best performances was chosen for the subsequent online session. The online session was conducted three times every 15 minutes for three hours, and the accuracy was calculated according to the results of each online session. The accuracy is defined in Equation (1), where *E* is the number of mistyped characters each time, *n* is the number of repetitions of the online test every 15 minutes, and *T* is the total number of characters used in each session. (In this case, *n* equals 3, and *T* equals 13.)

(1) $$\begin{array}{c} \text{Accuracy}=\frac{\sum_{i=1}^{n}\left(T-E_{i}\right)}{n\times T}\times 100\%. \end{array}$$

### 4.11. Steady-State Visual Evoked Potential (SSVEP) Stimulation Paradigm and Anti-Interference Index

Steady-state visual evoked potentials (SSVEP) are signals that are naturally generated by the brain at the same frequency of the flickering visual stimulus. This paradigm is one of the most commonly used technologies for BCI [54–57].

The same device described above was used for the SSVEP stimulus paradigm. A rectangle virtual square with 200 × 200 pixels was placed at the center of the monitor with a black background, and each steady-state visual stimulus was evoked by flickering this virtual square between black and white at a specific frequency for 2 s. (In this case, this frequency equals 10.5 Hz). A 2 s of resting period followed the steady-state visual stimulus, and the square was replaced with a white cross that did not flickering during this period. Each epoch consisted of one steady-state visual stimulus and one resting period, and ten epochs formed a trial that lasted 40 s (Figure S4).

This paradigm was modified from our previous work [58]. The software for the SSVEP stimulus display was developed with the MATLAB and Psychophysics Toolbox Version 3(PTB-3).

The same subject attended in this paradigm for two types of electrodes (a traditional wet electrode and the semidry electrode). The conditions of the SSVEP stimulation paradigm (location of the electrode, sampling rate, and electrode impedance of the traditional wet electrode) were the same as those for the previous mVEP stimulation paradigm. For each type of electrode, the SSVEP trial was conducted three times every fifteen minutes for three hours, and the data was acquired for further analysis.

The raw EEG data were digitally segmented into epochs from 300 ms before the onset of the steady-state visual stimulus and 2000 ms after the onset. The data in the range of -300 to 0 ms was set as the baseline. Then, a baseline correction was applied to the 2000 ms epochs. After that, Welch's method was applied to estimate the power spectral density (PSD) of the epochs with a time resolution of 2000 ms and frequency resolution of 0.5 Hz. Finally, PSD values of all of the epochs at the same time were averaged for further calculation. (In this case, there were 3 trials ×10 epochs within 15 minutes.)

To quantitatively analyze the performance of different types of electrodes over time, an anti-interference index was defined as the ratio of the PSD value at the signal frequency to that at the power line frequency. In this case, these were 10.5 Hz and 50 Hz, respectively. When the anti-interference index was higher, the performance was better.

### 4.12. Biocompatibility Test

A total of 2 healthy female Sprague-Dawley rats (mean age and weight 120 days and 210 g) were divided into three groups. The animals were kept in two plastic cages with access to food and water ad libitum. After the rats were anesthetized, the dorsal skin was shaved and disinfected. Four different points with the maximum interspace (30 mm) were selected on the back of the rats to paste and fix electrodes, including two AgPHMS, and two conventional Ag/AgCl gel-based electrodes as the control group. All electrodes were first fixed with athletic tape and then auxiliary fixed with nonwoven clothes. Particularly, we change the nonwoven clothes for each rat once a day.

## Supplementary Materials

Supplementary Materials Figure S1: XRD spectrum of the metalized melamine sponge and melamine sponge substrate. Figure S2: XPS peak-differentiation-imitating analysis of Ag 3d for the metalized melamine sponge. Figure S3: TGA (red) and differential thermal analysis (blue) of the metalized melamine sponge. Figure S4: stimulus and temporal scheme comparison of the mVEP BCI speller. Figure S5: (a) impedance change rate of the AgPHMS semidry electrode system on different channels. (b) Impedance change rate of the commercial wet electrode system on different channels. Figure S6: epoch of the SSVEP stimulus paradigm. Figure S7: (a) scalp after using the AgPHMS semidry electrodes. (b) Scalp after using a commercially available wet electrode. Supplementary Video 1: MVEP experiment: wireless typing the phrase “THUHELLO WORLD” by a brain-controlled virtual keyboard based on EEG mapping using AgPHMS semidry electrodes.
