# Supplementary material for: Ten-Hour Stable Noninvasive Brain-Computer Interface Realized by Semidry Hydrogel-Based Electrodes
Source: Research (Wash D C). 2022 Mar 10;2022:9830457. doi: 10.34133/2022/9830457 (PMC8933689; doi:10.34133/2022/9830457)
Supplement: Supplementary Materials — Figure S1: XRD spectrum of the metalized melamine sponge and melamine sponge substrate. Figure S2: XPS peak-differentiation-imitating analysis of Ag 3d for the metalized melamine sponge. Figure S3: TGA (red) and differential thermal analysis (blue) of the metalized melamine sponge. Figure S4: stimulus and temporal scheme comparison of the mVEP BCI speller. Figure S5: (a) impedance change rate of the AgPHMS semidry electrode system on different channels. (b) Impedance change rate of the commercial wet electrode system on different channels. Figure S6: epoch of the SSVEP stimulus paradigm. Figure S7: (a) scalp after using the AgPHMS semidry electrodes. (b) Scalp after using a commercially available wet electrode. Supplementary Video 1: MVEP experiment: wireless typing the phrase “THUHELLO WORLD” by a brain-controlled virtual keyboard based on EEG mapping using AgPHMS semidry electrodes. [file 9830457.f1.zip › Supporting Information .docx]

Supporting Information

**Ten-hour stable noninvasive brain-computer interface realized by semi-dry hydrogel-based electrodes**

Junchen Liu,^1,2^† Sen Lin,^2^† Wenzheng Li,^3^† Yanzhen Zhao,^1^ Dingkun Liu,^3^ Zhaofeng He,^4^ Dong Wang,^5^ Ming Lei,^2^* Bo Hong, ^3^* Hui Wu^1^*

^1^ State Key Laboratory of New Ceramics and Fine Processing, School of Materials Science and Engineering, Tsinghua University, Beijing 100084, China.

^2^ State Key Laboratory of Information Photonics and Optical Communications and School of Science, Beijing University of Posts and Telecommunications, Beijing 100876, China.

^3^ Department of Biomedical Engineering, School of Medicine, Tsinghua University, Beijing 100084, China.

^4^ School of Artificial, Beijing University of Posts and Telecommunications, Beijing 100084, China.

^5^ School of Biomedical Engineering, Hainan University, Haikou 570228, China

*Correspondence to: huiwu@tsinghua.edu.cn (Hui Wu), hongbo@tsinghua.edu.cn (Bo Hong), mlei@bupt.edu.cn (Ming Lei).

†These authors contributed equally to this work.

This file includes:

Supporting Information Figure 1-7, Supplementary Video 1.

**Supporting Information Figures**


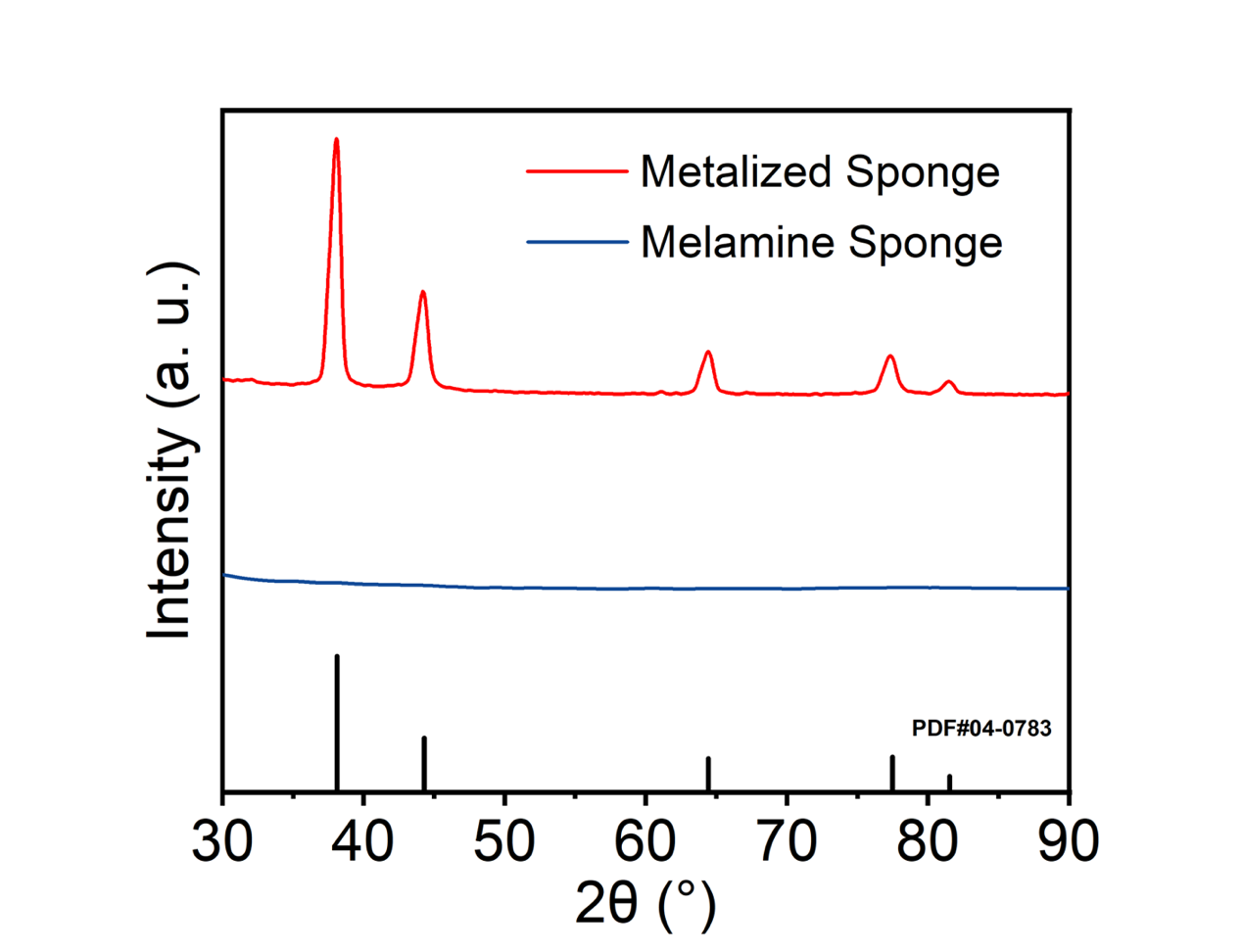


**Figure S1.** XRD spectrum of the metalized melamine sponge and melamine sponge substrate.


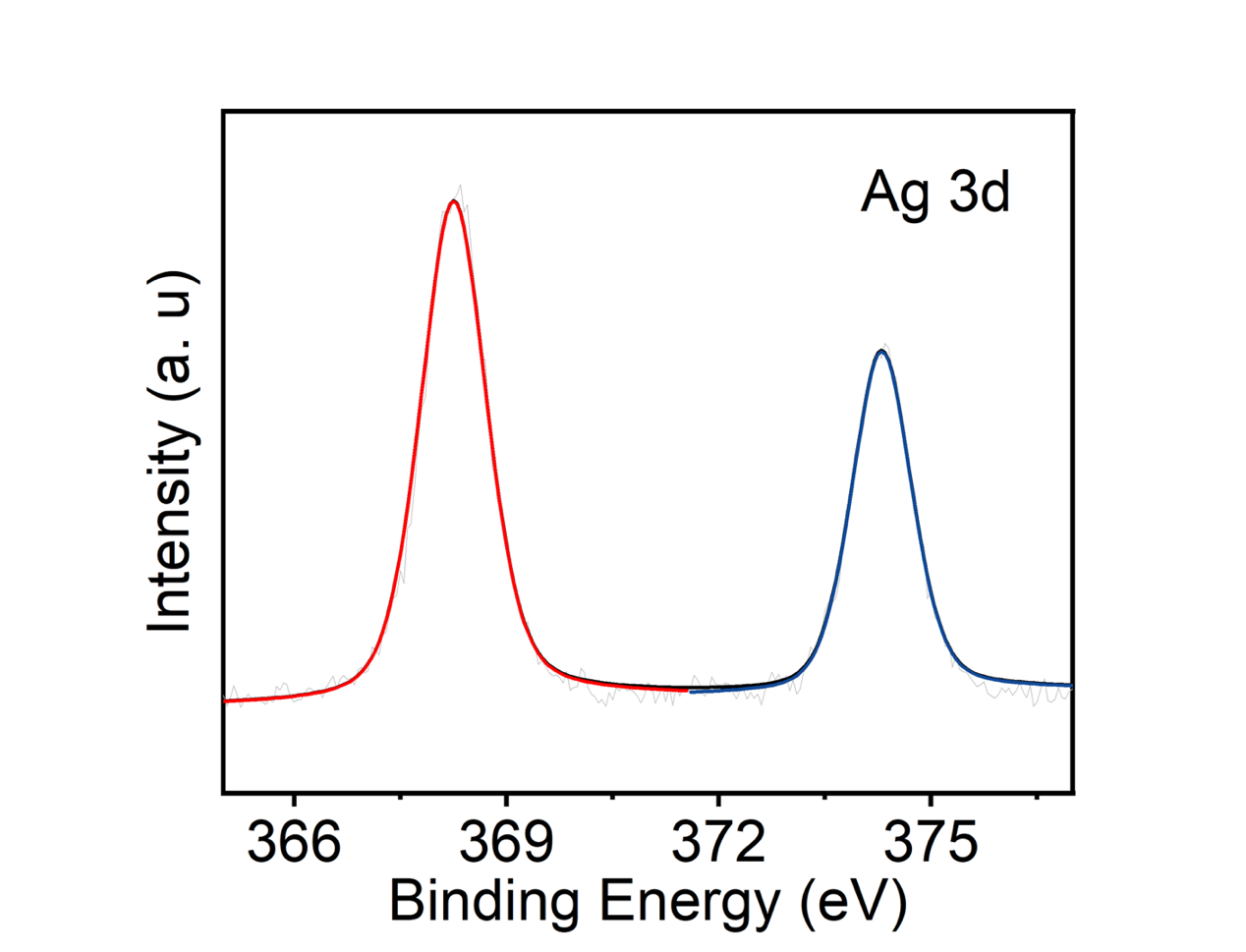


**Figure S2.** XPS peak-differentiation-imitating analysis of Ag 3d for the metalized melamine sponge.


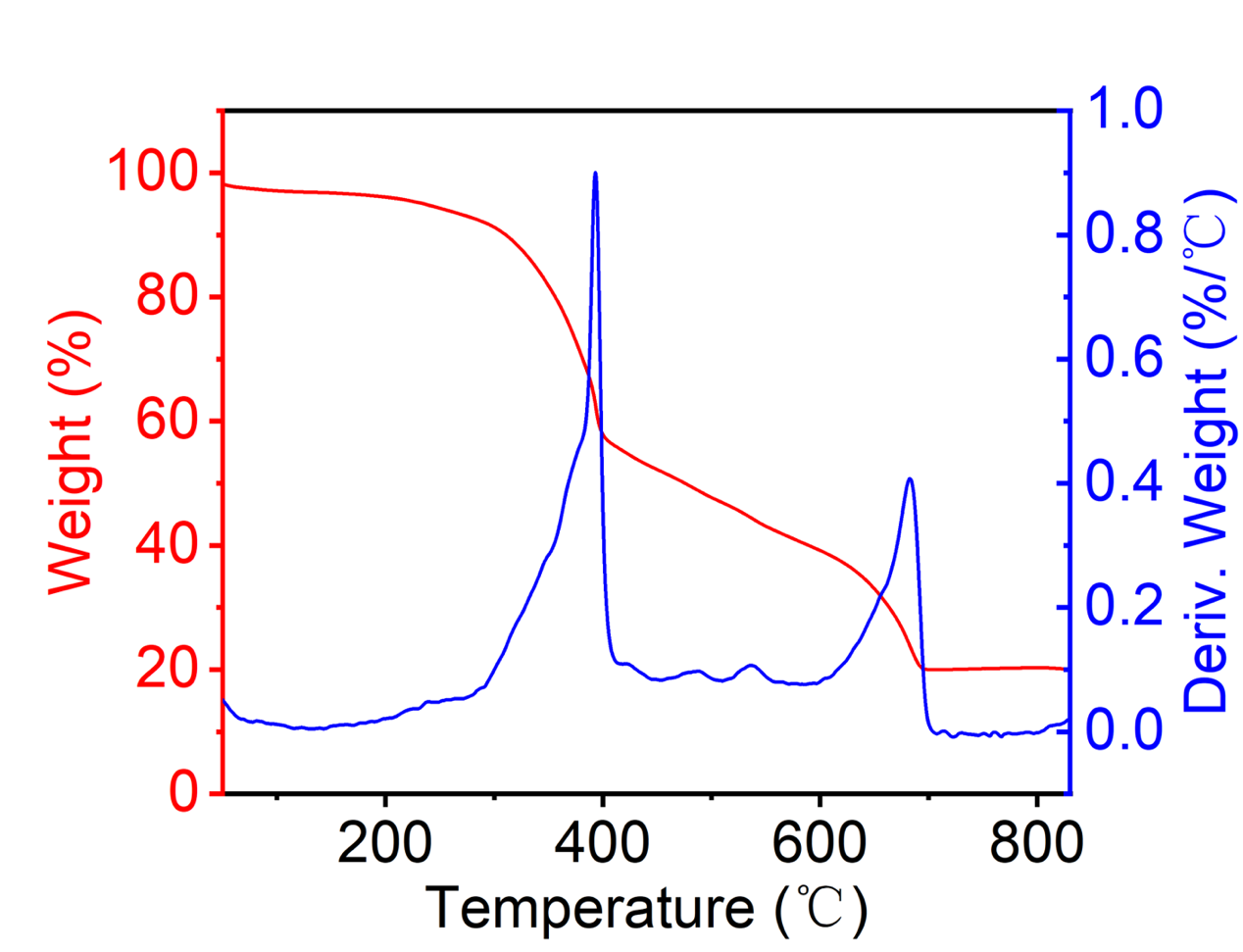


**Figure S3.** TGA (red) and differential thermal analysis (blue) of the metalized melamine sponge.


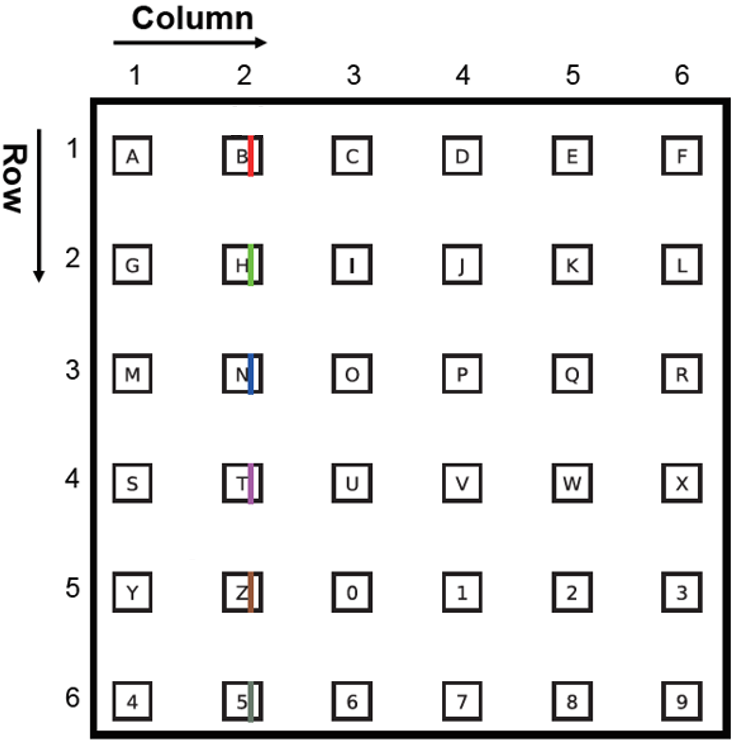


**Figure S4.** Stimulus and temporal scheme comparison of the mVEPs BCI speller.


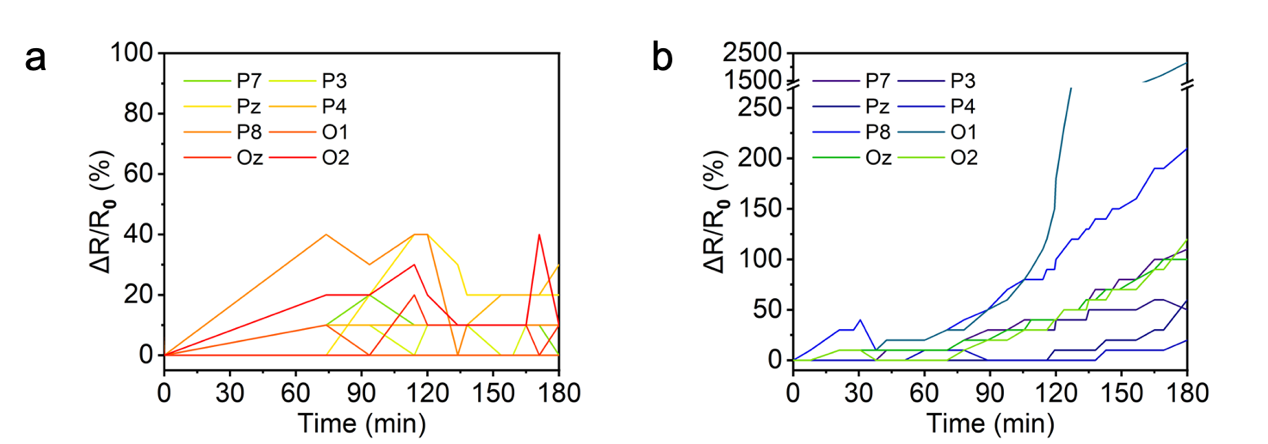


**Figure S5.** (a) Impedance change rate of the AgPHMS semi-dry electrode system on different channels. (b) Impedance change rate of the commercial wet electrode system on different channels.


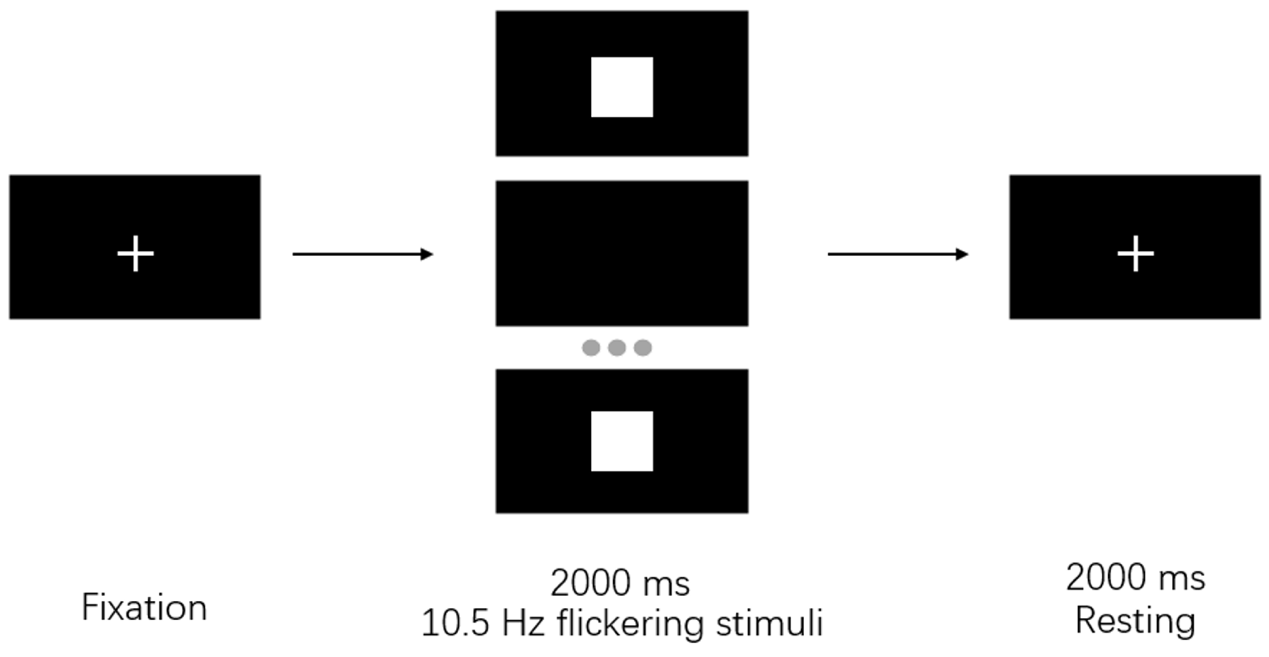


**Figure S6.** Epoch of the SSVEP stimulus paradigm.


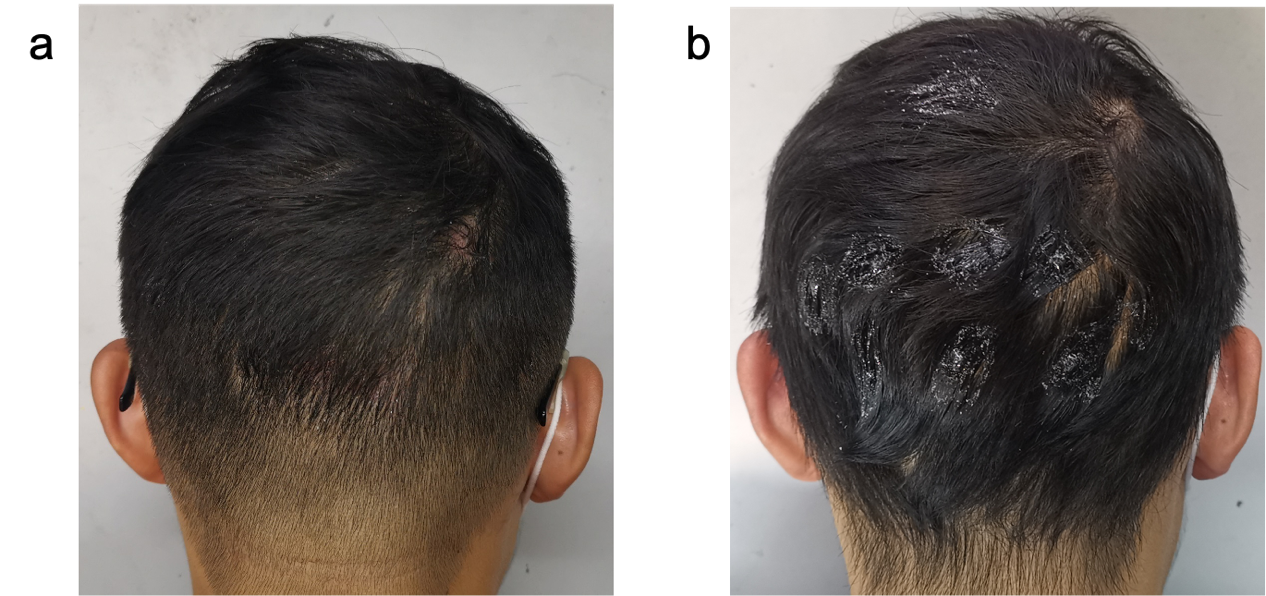


**Figure S7.** (a) Scalp after using the AgPHMS semi-dry electrodes. (b) Scalp after using a commercially available wet electrode.

**Supplementary Video**

**Supplementary Video 1.** MVEPs experiment: wireless typing the phrase “THUHELLO WORLD” by a brain-controlled virtual keyboard based on EEG mapping using AgPHMS semi-dry electrodes.
